# The relationship between test anxiety and emotion regulation: the mediating effect of psychological resilience

**DOI:** 10.1186/s12991-021-00360-4

**Published:** 2021-09-06

**Authors:** Yuguo Liu, Haiyan Pan, Runhuang Yang, Xingjie Wang, Jiawei Rao, Xingshan Zhang, Congcong Pan

**Affiliations:** School of Public Health, Guangdong Medical University, Dongguan, 523000 Guangdong China

**Keywords:** Test anxiety, Emotion regulation, Psychological resilience, Medical students, Academic performance

## Abstract

**Background:**

Test anxiety has been widely found in medical students. Emotion regulation and psychological resilience have been identified as key factors contributing to anxiety. However, studies on relationships were limited. This study investigated the links between psychological resilience, emotion regulation, and test anxiety in addition to exploring the differences about socio-demographic factors.

**Methods:**

A sample of 1266 medical students was selected through cross-sectional survey from a medical university in China during 2019. Data were obtained by network technique using designed questionnaire, which assesses the level of test anxiety, emotion regulation and psychological resilience, respectively.

**Results:**

Medical students experienced test anxiety at different levels, 33.7% of these were seriously. It revealed significant effects of the gender and academic performance on test anxiety. Results of logistic regression indicated that test anxiety was significantly associated with emotion regulation and psychological resilience (*p* < 0.01). Psychological resilience played a mediating role on the relationship between emotion regulation and test anxiety.

**Conclusions:**

These findings highlight the importance of psychological resilience and emotion regulation in understanding how psychological resilience relates to test anxiety in medical students. Resilience-training intervention may be developed to support students encountering anxiety during the exam.

**Supplementary Information:**

The online version contains supplementary material available at 10.1186/s12991-021-00360-4.

## Background

The term “test anxiety”, is considered as a series of psychological and behavioral responses when individual concern about possible failure on the exam or similar assessment situation [1]. It can occur at any phase of exam. According to psychologists and experts in education [2], an average level of anxiety is useful as an effective motivational factor can enhance one’s performance for more effort. While for some, taking excessive anxiety has an adverse effect on mental health and generates negative feelings for individuals, such as the sense of fear, stress, helplessness, anger, and so on [3]. Notably, test anxiety has a high prevalence all over the world [4–6].

Emotion regulation is defined as the process “by which individuals influence which emotions they have, when they have them, and how they experience and express these emotions” [7]. In general, cognitive reappraisal and expressive suppression are two commonly investigated strategies that have been associated with emotional responses and cognition processes [8]. According to the occurring time in which a strategy play a great role in the emotion regulation process, Gross [9] proposed that cognitive reappraisal emphasized antecedent-focused, referring to change the perception and assessment of emotional events and emotional consequence. And expressive suppression referred as a response-focused strategy that individual modify emotional responses through suppressing emotional expression, which appear in the late stage of emotion behaviors [9]. Previous studies [9–11] suggested that cognitive reappraisal, as an adaptive emotion regulation strategy, not only reduced negative emotion experience, but also decreased the sympathetic activity in the limbic brain system. Conversely, expressive suppression possibly was associated with negative emotion, even influenced physiological response including skin conductance level, and heart rate, and these symptoms might persist for some time [12].

Previous works have shown that test anxiety was regulated by psychological resilience [13, 14], which provided a new perspective to explore the intervention on test anxiety. Psychological resilience is recognized as the individual’s ability to effectively maintain psychological and physical health or positively adapt following exposure to the frustrations, difficulties, and adversities [15]. Most researchers study the assessment of two conditions referred in this concept of psychological resilience, including risk or adversity and positive adaptation. Beyond the above assessment, researchers also pay attention to understanding the protective factor of psychological resilience that ameliorate negative effects of threats to one’s functioning. Some studies show that psychological resilience plays a positive role in alleviating stress and arousing positive emotions [16]. In addition, individual with high level of psychological resilience might soon recover from negative emotional as well as bring the higher level of positive emotions [17].

Based on the above literature analysis, emotion regulation and psychological resilience are considered to be protective components for mental health and may have a synergistic effect on anxiety. If the above two factors are proved to influence test anxiety, their improvement could cause better outcomes in the prevention of test anxiety. To the best of our knowledge, there are few studies in this respect. Therefore, this study aims at a deeper understanding about the relation between test anxiety, psychological and emotion regulation. It may provide help to identify the psychological mechanism that lies behind test anxiety and to gain interventions about how effectively students deal with this negative emotion.

## Methods

### Study sample and procedure

The inclusion criteria were as follows: (1) undergraduate students at the Chinese medical university in Guangdong; and (2) respondents need to take an exam this semester. The exclusion criteria were as follows: (1) anxiety disorder; (2) have a history of other mental disorders; and (3) have a history of psychotropic drug use.

To estimate the sample size in the survey, a formula was taken about simple random sampling.

The sample size was calculated as follows:

$$n = \frac{{z^{2} \sigma^{2} }}{{d^{2} }},$$

where *n* is sample size, *z* is confident level, *d* is the error band and set to 0.2, *α* is estimate standard deviation = 3.8 (pretest). Setting the inspection level (*α*) as 0.05, *z* is 1.96. And the error band (*d*) is set to 0.2. Screening out research objects that do not meet the requirements, the sample size of the included study was finally determined to be 1300.

For avoiding sampling error, these students were told that the study about a test anxiety aimed at helping individuals. They recognized the value of study and signed informed consent during an initial investigations. Meanwhile, the present study was carried on the exam period from the first to second semesters in 2019 concerning acute anxiety evoked during stress was shown to be worse than usual [18]. Except for sampling error, the wording of questions in survey can result in bias in the collection of data. In order to avoid investigation error, investigators were conducted by uniform and standardized training.

Totally 1300 students were chosen from a medical university of China via simple random sampling in five majors (four in medicine, one in non-medicine). The study was approved by the institutional research ethics committee of Guangdong Medical University (Ref. YJYS2019027). Data were obtained from these participants who completed designed-questionnaires online after were informed of instructions by interview. It took about 15 min to finish responses on these questionnaires.

### Measures

Test Anxiety Scale (TAS) was developed in China by Wang [19], based on the Sarason’s Test Anxiety Scale [1]. The TAS is designed to explore the students how often they experience anxiety symptoms before, during, and after taking tests using 37 items. Participants were instructed to answer “Yes” or “No” for each item. If the answer is yes, one point will be counted, and if the answer is no, one point will not be counted, except six items are reversed (e.g., the 3rd, 15th, 26th, 27th, 29th, 33th). Respondents are divided into three different types of test anxiety according to TAS points. Scores greater than or equal to 12 are classified as low-test anxiety, scores falling above 12 and below 20 are classified as a normal level of anxiety, scores greater than or equal to 20 are classified as high level of test anxiety. The internal consistency as measured by Cronbach’s alpha in sample collected from Chinese was 0.77, and the split-half reliability was 0.60 [19]. The Cronbach’s alpha in this sample was 0.884, indicating an acceptable level of internal consistency.

The Emotion Regulation Questionnaire (ERQ) worked out by Gross and John [9] is one of the most widely used instruments for measuring emotion expression. This scale is designed to measure the emotions regulation of participants based on five universal emotions (including disgust, anger, sadness, fear and joy). This scale contains two dimensions, comprising six items each: expressive suppression (e.g., ‘Even when I am happy, I try not to show my feeling’, ‘When I am sad, I will suppress my emotion in order to not let others know how I really feel’), cognitive reappraisal (e.g., ‘I will try to revise my view about the surrounding people and thing to make myself happier’, ‘When faced with a situation that makes me angry, I change the way I look to relieve my anger’) and in addition to two items that whether an individual frequently uses an strategy (e.g., ‘I make sure not to express my emotions’, ‘I change the way I think to regulate my emotions’). Subjects respondent to items on a 7-point Likert scale from 1 (strongly disagree) to 7 (strongly agree), indicating that higher scores represent more frequently emotion regulation strategy used. Previous studies have shown that this scale has adequate reliability and validity, the Cronbach’s alpha (α) of cognitive reappraisal and expressive suppression was 0.83 and 0.77, respectively [20]. The Cronbach’s alpha of ERQ determined in this experiment was 0.903.

For assessment of psychological resilience among students, the Chinese adaptation of Connor–Davidson Resilience Scale (CD-RISC) was designed by researchers [21]. In creating the CD-RISC, Connor and Davidson [22] developed the scale that was designed to assess qualities, which was adapted by individuals in face of adversity. Basing on the analysis on data gained from both clinical and populations, the CD-RISC comprised 25 items including three dimensions model: strength, optimism and resilience. Respondents were asked to answer items based on their own situation. Each statement has a 5-point Likert scale anchored by “not true at all” and “true nearly all the time”. The CD-RISC has adequate internal consistency determined by the Cronbach’s alpha (*α* = 0.89) [23] and there is evidence shown that the reliable of the questionnaire items with college students samples in China was also adequate [21]. In the present study, we examined the internal reliability for the CD-RISC and it was adequate (Cronbach’s alpha = 0.955).

### Statistical analysis

Statistical analysis was performed using SPSS version 21.0 (SPSS Inc., USA). All study data for categorical variables were conducted by frequencies and proportions and data for continuous variables were conducted by means ± standard deviations (SDs). ANOVA and *t*-tests were used to identify the associated factors among test anxiety, resilience and emotion regulation. Comparisons the correlation between emotion regulation, resilience and test anxiety were performed via correlation analyses and the predictor of test anxiety was analyzed by regression analyses. Bootstrapping [24] was employed to examine the mediation effects of resilience on emotion regulation and test anxiety. Statistical significance was considered to be indicated by *p* < 0.05.

## Results

### General characteristics

A total of 1266 samples were further analyzed in this study, in which unqualified informations (e.g., too many same responses, answer is blank) had been removed. This scale had a response rate of 97.4%. All descriptive statistics for study participants are presented in Table 1. Of these participants, females held the predominant majority (*N* = 820, 64.8%). There were quite a few participants (78.3%) that took five to seven exams as the predominant majority. More than half of participants (83.9%) engaged in the club, 26.5% in part-time jobs. Most participants (61.7%) came from rural, while the rest (38.3%) were from city. More than two-thirds of participants (76.7%) were not from single-child family while only 23.3% were from single-child family. Maximum participants (47.8%) were first year, followed by 24.6% were third year.

**Table 1:** Socio-demographic characteristics of medical students (*N* = 1266)

Variable	Category	Frequency	Percentage
Gender	Male	446	35.2
	Female	820	64.8
Grade	First year	605	47.8
	Second year	264	20.9
	Third year	312	24.6
	Fourth year	64	5.1
	Fifth year	21	1.7
College	Public health	280	22.1
	Humanities and management	14	1.1
	Clinical medicine	100	7.9
	Medical technology	234	18.5
	Pharmacy	638	50.4
Part-time jobs	Yes	335	26.5
	No	931	73.5
Members of the club	Yes	1062	83.9
	No	204	16.1
Academic evaluation	Excellent	351	27.7
	Moderate	490	38.7
	Lower–moderate	241	19.0
	Poor	184	14.5
Examination number	Below 5	82	6.5
	5 to 7	991	78.3
	8 to 9	144	11.4
	Above 9	49	3.9
Birthplace	City	485	38.3
	Rural	781	61.7
Single-child family	Yes	295	23.3
	No	971	76.7

### Levels and associated factors of test anxiety, resilience and emotion regulation

In subjects group, 28.6% with mild level of test anxiety, 37.7% with moderate level of test anxiety, 33.7% with high level of test anxiety (Additional file 1). It showed that problematic test anxiety was prevalent among medical students. Meanwhile, students are more likely to opt for cognitive reappraisal compared with expressive suppression (Additional file 2).

The means and standard deviations of all study variables for test anxiety, resilience and emotion regulation are shown in Table 2. Additionally, gender and academic evaluation were significant factors using one sample *t*-test and one-way ANOVA, respectively (Table 2). Compared with female college students, the effects of psychological resilience and emotion regulation were much stronger, but the effects of test anxiety were much lower among male college students (*p* < 0.01, *p* < 0.01, *p* < 0.01, respectively). In terms of psychological resilience and emotion regulation, those who have excellent academic evaluation performed better than those who have poor academic evaluation, in contrast to the trend in test anxiety (*p* < 0.01, *p* < 0.05, *p* < 0.01, respectively).

**Table 2:** Means, standard deviations, and correlations (*N* = 1266)

Variable	Psychological resilience	Resilience	Strength	Optimism	Emotion regulation	Cognitive reappraisal	Expressive suppression	Test anxiety
Total	58.89 ± 15.28	29.68 ± 8.56	20.14 ± 5.01	9.07 ± 2.70	64.56 ± 11.92	34.30 ± 6.82	30.26 ± 6.82	16.75 ± 7.62
Gender								
Male	60.96 ± 17.58	30.99 ± 9.85	20.64 ± 5.71	9.33 ± 2.97	65.52 ± 13.93	33.97 ± 7.72	31.55 ± 7.63	15.77 ± 7.74
Female	57.77 ± 13.76	28.97 ± 7.68	19.86 ± 4.56	8.93 ± 2.53	64.03 ± 10.63	33.48 ± 6.28	29.55 ± 7.61	17.27 ± 7.51
*T*	3.33**	3.75**	2.49*	2.39*	1.97*	− 1.184	4.66**	− 3.36**
Grade								
First year	59.87 ± 15.31	30.19 ± 8.61	20.45 ± 4.97	9.22 ± 2.69	64.42 ± 11.99	34.22 ± 6.88	30.20 ± 7.25	16.61 ± 7.61
Second-year	59.59 ± 16.70	30.36 ± 9.09	20.10 ± 5.47	9.13 ± 2.92	64.91 ± 12.54	34.26 ± 6.88	30.65 ± 7.17	16.74 ± 7.74
Third year	56.26 ± 13.83	28.10 ± 7.83	19.43 ± 4.69	8.73 ± 2.52	64.54 ± 11.45	34.35 ± 6.69	30.19 ± 6.61	17.02 ± 7.74
*F*	3.17*	3.80**	2.38*	1.18	0.26	0.14	0.81	0.17
Academic evaluation								
Excellent	61.89 ± 13.41	31.22 ± 7.59	21.26 ± 4.48	9.40 ± 2.46	65.89 ± 11.08	35.28 ± 6.21	30.61 ± 6.81	15.49 ± 6.67
Moderate	60.22 ± 14.41	30.37 ± 8.14	20.56 ± 4.61	9.29 ± 2.67	64.76 ± 11.46	34.55 ± 6.57	30.21 ± 7.00	16.51 ± 7.61
Lower–moderate	56.40 ± 14.73	28.37 ± 8.23	19.27 ± 4.90	8.77 ± 2.58	63.46 ± 11.67	33.55 ± 6.96	29.91 ± 6.61	16.85 ± 7.94
Poor	52.92 ± 19.11	26.64 ± 10.62	18.00 ± 6.17	8.28 ± 3.15	62.92 ± 14.47	32.75 ± 8.01	30.17 ± 8.12	19.64 ± 8.22
*F*	17.93**	15.01**	21.73**	9.22**	3.37*	6.86**	0.51	12.48**
Single-child family								
Yes	59.37 ± 16.45	29.94 ± 9.14	20.19 ± 5.35	9.24 ± 2.85	64.01 ± 12.75	33.49 ± 7.16	30.52 ± 7.41	17.12 ± 7.88
No	58.75 ± 14.92	29.61 ± 8.37	20.12 ± 4.90	9.02 ± 2.65	64.73 ± 11.65	34.54 ± 6.70	30.18 ± 6.93	16.63 ± 7.54
*T*	0.58	0.58	0.21	1.21	− 0.90	− 2.32*	0.72	0.95
Part-time jobs								
Yes	60.71 ± 15.44	30.84 ± 8.72	20.52 ± 5.02	9.35 ± 2.75	66.00 ± 12.75	34.90 ± 6.97	31.10 ± 7.30	16.37 ± 7.97
No	58.24 ± 15.18	29.26 ± 8.46	20.00 ± 5.00	8.98 ± 2.68	64.04 ± 11.56	34.08 ± 6.76	29.95 ± 6.93	16.88 ± 7.49
*T*	2.54*	2.91**	1.62	2.18*	2.59*	1.88	2.56*	− 1.05
Members of the club								
Yes	59.26 ± 14.79	29.86 ± 8.26	20.27 ± 4.88	9.12 ± 2.65	64.68 ± 11.59	34.46 ± 6.67	30.22 ± 6.94	16.86 ± 7.55
No	57.00 ± 17.53	28.74 ± 9.91	19.42 ± 5.60	8.84 ± 2.93	63.92 ± 13.49	33.47 ± 7.53	30.45 ± 7.59	16.17 ± 7.97
*T*	1.73	1.53	2.03*	1.34	0.83	1.90	− 0.43	1.18
Birthplace								
City	59.32 ± 15.94	29.86 ± 8.84	20.17 ± 5.23	9.29 ± 2.82	64.74 ± 12.27	34.09 ± 6.78	30.66 ± 7.34	17.41 ± 7.87
Country	58.63 ± 14.87	29.57 ± 8.38	20.12 ± 4.87	8.94 ± 2.62	64.44 ± 11.70	34.43 ± 6.85	30.01 ± 6.85	16.33 ± 7.44
*T*	0.78	0.57	0.18	2.23*	0.44	− 0.87	1.58	2.46*

^*^p < 0.05; **p < *0.01*

The correlation analysis results indicated that test anxiety has a significant negative correlation with psychological resilience (*r* = − 0.382, *p* < 0.01) and emotion regulation (*r* = − 0.158, *p* < 0.01) (Table 3). Meanwhile, the two dimensions of emotion regulation and the three dimensions of psychological resilience were significantly negative correlations with test anxiety, respectively. In addition, the relation between emotion regulation and psychological resilience is a significant positive correlation (*r* = 0.507, *p* < 0.01).

**Table 3:** The correlation coefficients for the test anxiety, emotion regulation and resilience (*N* = 1266)

Variable	1	2	3	4	5	6	7	8
1. Test anxiety								
2. Emotion regulation	− 0.158**							
3. Cognitive reappraisal	− 0.191**	0.854**						
4. Expressive suppression	− 0.082**	0.864**	0.476**					
5. Resilience	− 0.382**	0.507**	0.559**	0.316**				
6. Resilience	− 0.374**	0.500**	0.537**	0.326**	0.972**			
7. Strength	− 0.364**	0.492**	0.562**	0.288**	0.946**	0.867**		
8. Optimism	− 0.304**	0.371**	0.420**	0.221**	0.827**	0.723**	0.723**	

^*^p< 0.05; **p < *0.01*

### Testing the mediating role of resilience

Analysis was performed to find whether psychological resilience and emotion regulation has an effect on test anxiety. The multiple regression analysis results indicated that cognitive reappraisal of emotion regulation negatively predicted test anxiety (*β* = − 0.191, *p* < 0.01) (Table 4). Similarly, as a predictor, resilience and strength negatively predicted test anxiety (*β* = − 0.235, *p* < 0.01; *β* = -0.160, *p* < 0.01, respectively).

**Table 4:** The regression analyses for the test anxiety, emotion regulation and resilience (*N* = 1266)

Independent variable	Equation 1 (dependent variables: test anxiety)				Equation 2 (dependent variables: test anxiety)			
	B^a^	SE^b^	*β*^c^	*t*	B	SE	*β*	*t*
Constant	24.046	1.079		22.290**	27.879	0.821		33.941**
Cognitive reappraisal	− 0.213	0.031	− 0.191	− 6.900**				
Resilience					− 0.209	0.046	− 0.235	− 4.506**
Strength					− 0.244	0.079	− 0.160	− 3.078
*R*^*2* d^				0.036				0.146
*F*				47.611**				108.230**

**p < 0.01, which present significant differences

^a^Regression coefficient was presented in B

^b^Standard error was presented in SE

^c^Standardized regression coefficient was presented in *β*

^d^Coefficient of determination was presented in *R*^*2*^

Furthermore, regression analyses found that dimensions of psychological resilience might be taken to have a mediating role. To test the mediation, total effects model was built by taking cognitive reappraisal as independent variable *X*, resilience and strength as mediating variables M1 and M2, respectively, and test anxiety as dependent variable *Y*. The bootstrapping method was adopted to examine mediation effects and 95% confidence interval (CI) level was used. When the value calculated by CI did not include 0, it could be determined that mediating effects have occurred. Results presented that the *ab* value was statistically significant, indicating that psychological resilience played a mediating role between emotion regulation and test anxiety. Therefore, the constructed model about emotion regulation—resilience—test anxiety was fit. The indirect effect of resilience between emotion regulation and test anxiety did not include 0 (95% CI − 0.189, − 0.091) (Table 5), the indirect effect of strength between emotion regulation and test anxiety also did not include 0 (95% CI − 0.162, − 0.048) (Table 5), indicating resilience and strength as mediators. And on this basis the mediating variables being controlled, it was found that the direct effect of cognitive reappraisal on test anxiety included 0 (95% CI − 0.027, 0.112). These results further suggest that resilience and strength performed a complete mediating role between emotion regulation and test anxiety.

**Table 5:** Mediation model of resilience between emotion regulation and test anxiety (*N* = 1266)

Roadmap	Category	Effect ratio	95% CI (lower, upper)	Relative mediation effect (%)
Cognitive reappraisal → test anxiety	Total effect	− 0.213	− 0.273, − 0.152	100
Cognitive reappraisal → test anxiety	Direct effect	0.043	− 0.027, 0.112	14.40
Cognitive reappraisal → resilience → test anxiety	Indirect effect	− 0.146	− 0.189, − 0.091	48.76
Cognitive reappraisal → strength → test anxiety		− 0.110	− 0.162, − 0.048	36.84

## Discussion

### The population characteristics of medical students’ test anxiety, emotion regulation, and psychological resilience

In China, high test anxiety has become the serious health problem to disturb the college students with the ascending tendency, which increased from 27.52 [25] to 35.7% [26] for nearly a decade. The present cross-sectional study conducted in a medical university among the undergraduate students showed that the test anxiety was prevalent, even more (33.7%) of the students experienced unhealthy test anxiety. The results were consistent with recent trends. Overall, summaries of test anxiety research from around the world have shown that students who major in medicine tend to have higher test anxiety compared with other majors. That is partly because the subjects with future careers related to human life take more time to pass the exam because of strict requirements for knowledge and skills [4, 6, 27].

In the present study, we found interesting results with regard to the effects of academic performance and gender which were the main focus on the test anxiety. Many researches have provided strong support for the fact that women in trouble tend to adopt negative emotion regulation and have significantly higher levels of the cognitive component on anxiety than man, due to their emotional and psychological characteristics [28, 29]. The academic performance findings suggested that students with excellent academic performance have significantly higher levels of the emotion regulation and psychological resilience, and were less affected by test anxiety. This was in line with previous findings that there was a negative relation between test anxiety and educational achievement [30]. In part, students with excellent academic performance have developed learning abilities and strategies to easily cope with examinations, while poor students suffered from unrealistic expectations which may increase excessive anxiety in exam. Although the present findings may be considered preliminary, it suggests that individual differences on test anxiety should be regarded with some care in practice.

### The relationship between emotional regulation and test anxiety

Emotional regulation is a process in which individuals consciously manage and change their emotions, as well as closely related to mental health. The study found that emotion regulation had a significantly negative correlation with test anxiety, and test anxiety was negatively predicted by emotion regulation. These findings were consistent with previous studies. In both Moltrecht [31] and Aldao [32] studies, emotional regulation can reduce the anxiety level of individuals caused by stressful events, and promote good psychological adaptation of individuals. These results revealed that students who adopt the emotion regulation strategy will have more positive and healthy mental state, which is conducive in reducing the occurrence of exam anxiety.

In addition, the results showed students chose more cognitive reappraisal in the adoption of emotion regulation strategy. Interestingly, the situation is similar in different cultural background. Studies with college students in America, Australia, and Belgium [33, 34] found that subjects believed cognitive reappraisal can improve anxiety to mental flexibility. That may be because the cognitive reappraisal strategy with long-term effects is more adaptive in emotion regulation strategy selection than expressive suppression. Some research point out that adolescents with anxiety disorders can reduce their negative emotion after using cognitive reappraisal [35–37].

To cultivate positive emotions and cope with negative emotions (anxiety), active intervention is necessary before emotional reaction, such as choosing favorable situations, cognitive reappraisal strategies.

### The relationships between test anxiety, emotion regulation, and psychological resilience

Recent studies have indicated that test anxiety makes the individual prone to negative emotions (e.g., the sense of fear, stress, helplessness and anger) [3, 38], even more leads to the emotion disorders and subsequent psychological problems in groups [39]. However, extant research has shown that test anxiety, emotion regulation and psychological resilience are related, but has yet to examine the interaction with each other. The present study will address this gap.

As previously expected, there is a significant negative correlation between test anxiety and psychological resilience. There is a significant positive correlation between emotion regulation and psychological resilience. These results were consistent with previous studies [40–42], which reported that psychological resilience is associated with positive emotions and might be used to predict anxiety. Moreover, several recent studies have indicated psychological resilience as the protective factor can cope with the negative emotion, which arises in individuals in the face of adversities or anxiety [43]. Based on this finding, the mediating effect analysis was used in the current study to examine the relationship between test anxiety, emotion regulation, and psychological resilience. Consequently, a relationship between emotion regulation and test anxiety acts via the psychological resilience. Much of the recent work in anxiety [13, 14], psychological resilience has been focused more and more by researchersbecause of its protective effect on anxiety. These findings provided initial evidence that psychological resilience may have positive effects on emotion regulation working on a test anxiety. This may provide insight for clinical psychologists on cultivating the psychological resilience to alleviate test anxiety among medical students.

## Conclusions

The aim of this study was a deeper understanding about the relation between test anxiety, emotion regulation, and psychological resilience to identify the psychological mechanism that lies behind test anxiety and to gain anxiety interventions about how effectively students deal with this emotion. Our empirical research suggests that emotion regulation indirectly affected test anxiety through the mediating effect of psychological resilience. However, investigation on the continuous psychological development of subjects was limited due to the cross-sectional study design. The background of the subjects and irrelevant variables that could impair data reliability, such as environmental factors, need to be explored further to improve reliability in future research.

## Supplementary Information

**Additional file 1.** Prevalence of test anxiety among medical students (*N* = 1266).

**Additional file 2.** Descriptive analysis of emotion regulation among medical students (*N* = 1266).

## Data Availability

The datasets analyzed during the current study are available from the corresponding author on reasonable request.

## Abbreviations

TAS: Test Anxiety Scale
ERQ: Emotion Regulation Questionnaire
CD-RISC: Connor–Davidson Resilience Scale
CI: Confidence interval
