# Supplementary material for: The relationship between test anxiety and emotion regulation: the mediating effect of psychological resilience
Source: Ann Gen Psychiatry. 2021 Sep 6;20:40. doi: 10.1186/s12991-021-00360-4 (PMC8419945; doi:10.1186/s12991-021-00360-4)
Supplement: Supplementary file 1 — Additional file 1. Prevalence of test anxiety among medical students (N = 1266). [file 12991_2021_360_MOESM1_ESM.docx]

Additional file 1:

Prevalence of test anxiety among medical students (*N* = 1266).

| Prevalence | Means | Standard deviations | Percentage (%) |
| --- | --- | --- | --- |
| Mild level of anxiety | 8.01 | 2.039 | 28.6 |
| Moderate level of anxiety | 15.48 | 2.244 | 37.7 |
| High level of anxiety | 25.57 | 4.321 | 33.7 |
| Test anxiety (total) | 16.75 | 7.623 | 100 |
