# Supplementary material for: The relationship between test anxiety and emotion regulation: the mediating effect of psychological resilience
Source: Ann Gen Psychiatry. 2021 Sep 6;20:40. doi: 10.1186/s12991-021-00360-4 (PMC8419945; doi:10.1186/s12991-021-00360-4)
Supplement: Supplementary file 2 — Additional file 2. Descriptive analysis of emotion regulation among medical students (N = 1266). [file 12991_2021_360_MOESM2_ESM.docx]

| Factor | Mean | Standard deviations |
| --- | --- | --- |
| cognitive reappraisa | 34.30 | 6.824 |
| expressive suppression | 30.26 | 7.046 |
| emotion regulation | 64.56 | 11.916 |

Additional file 2:

Descriptive analysis of emotion regulation among medical students (*N* = 1266).
